# Clinical Notes on Uterine Surgery

**Published:** 1886-03

**Authors:** 


Clinical Notes on Uterine Surgery with special reference to the Management of the Sterile Condition. By J. Marion Sims, A. B., M. D., late Surgeon to the Woman's Hospital, etc. Memorial Edition. New York: Wm. Wood & Co., 1886.

This work is doubtless well known to every practitioner as Marion Sims' sole contribution to the standard literature of medicine. Although a frequent contributor to the current medical periodicals, he had, as he himself confesses, "an innate horror of writing," by reason of which the greater part of his vast stores of knowledge and experience was not given to the profession at large, but was handed down by a species of oral tradition to those who enjoyed the privilege of association with him. Partly from this fact and partly from the atmosphere of jealousy and coldness with which he was surrounded in his adopted city up to the day of his death, his views in regard to the treatment of dysmenorrhœa and sterility, as set forth in this volume, have never met with the recognition which they deserved. At the outset they encountered the powerful influence of the bitter opposition of Emmet, who, since Sims' death, has stigmatized the operation of division of the cervix as malpractice, although neither he nor any other author has ever offered any statistics which would sustain this view. On the contrary, those surgeons who have been able to emancipate themselves from the dominant influence of what may be styled the Emmet school of gynæcology have confirmed the good results which were obtained by Sims in his mode of treatment. Yet so small is the number of such surgeons that the operation of division of the cervix, one of the most satisfactory in the whole realm of gynæcology, has unfortunately now become almost a lost art. If the issue of this memorial edition shall result in calling attention anew to the methods of treatment detailed in this work of the great "father of gynæcology," the publishers will have merited the lasting gratitude of the medical profession and of their suffering patients of the gentler sex.

